# Recent Advancements in the Development of Anti-Breast Cancer Synthetic Small Molecules

**DOI:** 10.3390/molecules26247611

**Published:** 2021-12-15

**Authors:** Eslam B. Elkaeed, Hayam A. Abd El Salam, Ahmed Sabt, Ghada H. Al-Ansary, Wagdy M. Eldehna

**Affiliations:** 1 Department of Pharmaceutical Sciences, College of Pharmacy, AlMaarefa University, Ad Diriyah, Riyadh 13713, Saudi Arabia; ikaeed@mcst.edu.sa; 2 Department of Green Chemistry, National Research Center, Dokki, Cairo 12622, Egypt; hayam_nrc@yahoo.com; 3 Chemistry of Natural Compounds Department, National Research Center, Dokki, Cairo 12622, Egypt; sabt.nrc@gmail.com; 4 Department of Pharmaceutical Chemistry, Faculty of Pharmacy, Ain Shams University, Cairo 11566, Egypt; ghada.mohamed@pharma.asu.edu.eg; 5 Department of Pharmaceutical Chemistry, Faculty of Pharmacy, Kafrelsheikh University, Kafrelsheikh 33516, Egypt

**Keywords:** breast cancer therapy, organic synthesis, chemical scaffold, mechanistic insights, human malignancies

## Abstract

Among all cancer types, breast cancer (BC) still stands as one of the most serious diseases responsible for a large number of cancer-associated deaths among women worldwide, and diagnosed cases are increasing year by year worldwide. For a very long time, hormonal therapy, surgery, chemotherapy, and radiotherapy were used for breast cancer treatment. However, these treatment approaches are becoming progressively futile because of multidrug resistance and serious side effects. Consequently, there is a pressing demand to develop more efficient and safer agents that can fight breast cancer belligerence and inhibit cancer cell proliferation, invasion and metastasis. Currently, there is an avalanche of newly designed and synthesized molecular entities targeting multiple types of breast cancer. This review highlights several important synthesized compounds with promising anti-BC activity that are categorized according to their chemical structures.

## 1. Introduction

Worldwide, breast cancer is the most prevalent cancer and the second type of cancer leading to mortality in women [1]. More than 276,480 new breast cancer cases are expected to be identified in women in the United States in 2020 [2]. Risk factors linked to breast cancer continue to be identified [3]. Accordingly, breast cancer is considered one of the urgent public health problems globally [4].

Breast cancer is promoted via different types of factors (endogenous and exogenous) which have differing outcomes [5]. Treatment of invasive BC is quite challenging due to its aggressive features [6,7]. A plentiful body of evidence suggests that long-term survival can be increased, if there is an ability to suppress distant metastasis [8,9]. Breast cancer treatment depends on a mechanism(s) that can be targeted by natural or synthetic compounds that already exist as antitumor drugs [10,11]. Not surprisingly, literature reviews revealed that many heterocyclic and/or other scaffolds have been developed as anti-breast cancer agents [12,13,14].

### Different Subtypes of Breast Cancer

**Figure 1:**
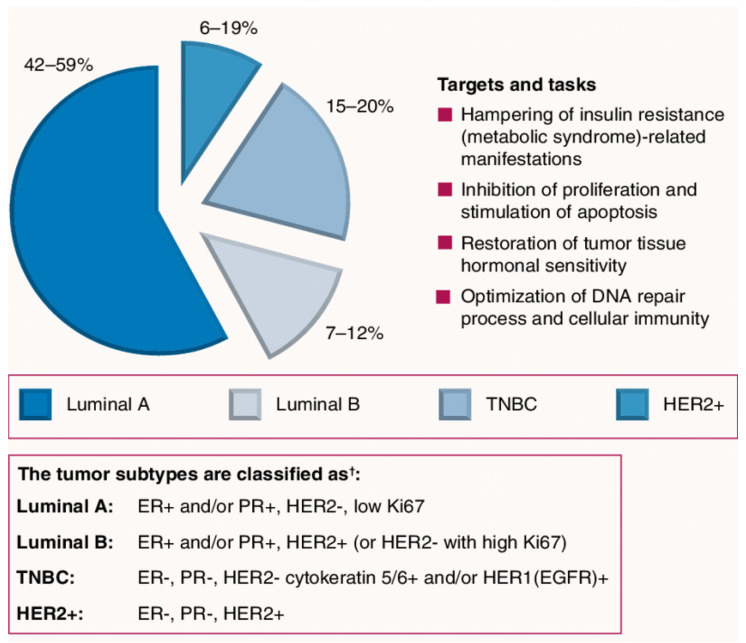
Representation for different breast cancer subtypes.

There are four main subtypes of female breast cancer [15] (Figure 1):

Luminal A: This group has tumors that are estrogen-receptor and/or progesterone-receptor positive (+ve ER and/or +ve PR), but it is human epidermal growth factor receptor negative (−ve HER2). In addition, this type possesses protein Ki-67 in low levels, which is beneficial to control the growth of cancer cells. This type generally has the best prognosis with low grade and grows slowly.
Luminal B: This group has tumors that are positive for ER and HER2 but negative for PR and possesses protein Ki-67 in high levels. Luminal B BCs mainly grow faster than luminal A BCs, and their prognosis is slightly worse.
  HER2-enriched (HR−/HER2+): This group has tumors that are negative ER, PR and positive for HER2. It is treated with targeted therapy to target the HER2 protein although it grows faster than luminal cancers with a worse prognosis. Examples of targeted therapies include Pertuzumab, Trastuzumab Deruxtecan, Trastuzumab Emtansine, Lapatinib, Trastuzumab, and Neratinib.
  Basal-like: This group is also named triple-negative breast cancer; it is negative for ER, PR and HER2. Unfortunately, it is the most common type with women with the gene mutation of BRCA1 among young and African-American women, and it is characterized by the missing signature of three biomarkers (PR, ER, HER2 proteins). Accordingly, it is the most aggressive type of BC.

There are six subtypes of basal-like breast cancer reported by Lehmann et al. [16] which are treated differently [17]: basal-like 1 (BL1), basal-like 2 (BL2), Immunomodulatory (IM), Mesenchymal (M), Mesenchymal stem like (MSL), Luminal androgen receptor (LAR).

The BC subtype HR+/HER2− (HR+ stands for BC cells possessing receptors for the hormones estrogen or progesterone) has been identified as the most common subtype with a rate of 85.8 new cases per 100,000 women, according to 2012–2016 cases. This is a rate more than 6-fold higher than the TNBC rate of 13.0 and the HR+/HER2+ BC rate of 12.9, and over 15-fold higher than HR−/HER2+ BC rate of 5.4.

## 2. Conventional Treatment and Its Drawbacks

Management of breast cancer involves many treatment approaches that comprise chemotherapy, radiotherapy, surgery, and hormonal therapy [18]. Early stages of breast cancer, I and II, can be effectively treated by radiotherapy and chemotherapy, sparing the breast tissue [19]. These stages are accompanied by multiple forms of diseases such as pericarditis, rib fracture, tissue necrosis, brachial plexopathy, in addition to the second non-breast malignancies [20]. 

Multidrug resistance stands as a stubborn obstacle for management of BC which ultimately leads to death. Accordingly, the urgency arises for the thorough understanding and analysis of the cancer molecular basis of resistance and tailoring new drugs that target it effectively [21].

The emergence of resistance is caused by different mechanisms. Modulation of the drug efflux membrane transporter is the primary culprit for such resistance; these include P-glycoprotein, MRP 1, and BCRP [22,23]. This is evidenced by the observed patients’ immunity to several previously effective antineoplastic agents such as anthracyclines (epirubicin, daunorubicin, doxorubicin and mitoxantrone), taxanes (docetaxel, paclitaxel), and capecitabine [24]. Furthermore, the practice of using mono therapy in the treatment of breast cancer gave a remarkable capacity to start flooding the tumor mass with a growing supply of new cancer cells [25]. Moreover, the reduction in white blood cells and red blood cells as major side effects for chemotherapy led to increasing the risk of infection and anemia with decreased O_2_ carrying capacity for the cells, respectively [26]. Accordingly, seeking an alternative approach for the management of BC on the basis of all these understandings is an urgent necessity which could prevent and minimize the risk of unexpected side effects of conventional treatment methods [27].

## 3. Recent Drugs Approved for Breast Cancer Management

Many drugs are approved by the FDA for various BC subtypes therapy, and are described in the following sections.

### 3.1. Eribulin

**Figure 2:**
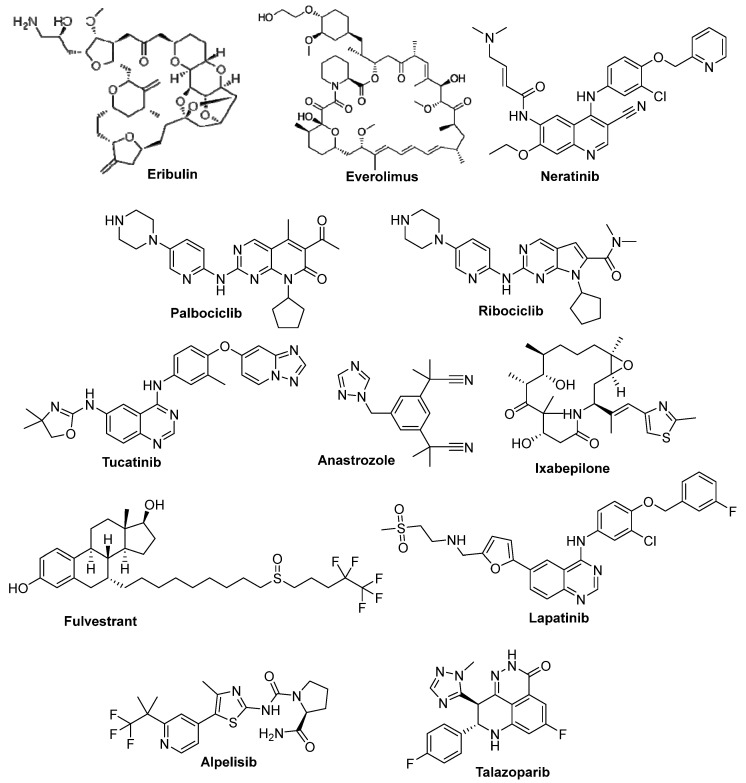
FDA-approved drugs for the management of different human breast malignancies.

Eribulin (Figure 2) is a synthetic ketone analog of the macrocyclic chemotherapeutically active halichondrin B derived from the sea sponge *Halichondria okadai*. Eribulin is a potent mitotic inhibitor with a unique mechanism of action as an inhibitor of microtubule dynamics. Eribulin received its FDA approval in 2010 to manage the metastatic breast cancer in patients who have received at least two prior chemotherapy regimens for late-stage disease [28]. 

### 3.2. Everolimus

Everolimus (Figure 2), a derivative of the natural macrocyclic lactone sirolimus, is an orally bioavailable inhibitor of mTOR. On 20 July 2012, the U.S. FDA approved Everolimus for the treatment of post-menopausal women with advanced HR+/HER2− breast cancer. It is worth mentioning that Everolimus has been approved also for tuberous sclerosis complex-associated partial-onset seizures (in 2018), progressive, nonfunctional gastrointestinal and lung neuroendocrine tumors (in 2016), and advanced pancreatic neuroendocrine tumors (in 2011), as well as being the first approved pediatric-specific dosage form for the management of a rare pediatric brain tumor called subependymal giant cell astrocytoma [29,30,31].

### 3.3. Neratinib

Neratinib (Figure 2), a 4-anilinoquinoline-based orally bioavailable kinase inhibitor, is an irreversible pan-ErbB inhibitor of EGFR, HER2, and HER4 targeting the intracellular domain, which results in reduced phosphorylation and downstream pathways activation. Neratinib has been recently FDA- and EMA-approved for the extended adjuvant treatment of early stage HER2-positive BC [32].

### 3.4. Palbociclib

Palbociclib (Figure 2) is a pyrido[2,3-*d*]pyrimidin-7-one derivative which acts as a selective CDK4/6 inhibitor. On 3 February 2015, the FDA granted accelerated approval of Palbociclib (in combination with letrozole) for postmenopausal women with advanced breast cancer, then in 2016 Palbociclib was approved for treatment of HR+/HER2− metastatic BC. Notably, Palbociclib was the first-in-class CDK 4/6 inhibitor approved by the FDA [33].

### 3.5. Ribociclib

Ribociclib (Figure 2) is a pyrrolo[2,3-*d*]pyrimidine-based potent selective inhibitor of cyclin-dependent kinases (CDKs) 4 and 6. In 2017, the FDA approved Ribociclib for treatment of patients with HR+/HER2− metastatic BC. One year later, it was additionally approved for HR+/HER2− advanced breast cancer [34].

### 3.6. Tucatinib

Tucatinib (Figure 2) is an orally bioavailable HER2 tyrosine kinase inhibitor. On 17 April 2020, the FDA granted an approval for tucatinib, in combination with Trastuzumab and capecitabine, for the management of patients with advanced unresectable or metastatic HER2-positive BC [35].

### 3.7. Anastrozole

Anastrozole (Figure 2) is a reversible, nonsteroidal inhibitor of the aromatase enzyme that is taken orally. Anastrozole is an essential anticancer drug that is indicated as an adjuvant remedy in the treatment of HR+ BC in postmenopausal women at early stages [36].

### 3.8. Ixabepilone

Ixabepilone (Figure 2), a semisynthetic analog of epothilone B, stabilizes the microtubules which are essential for cell division via suppression of the dynamic of microtubules (aβ-II and aβ-II). Hence, it arrests the cell cycle in the G2-M phase, induces tumor cell apoptosis and inhibits tumor cell proliferation. In 2007, Ixabepilone was granted an FDA approval for the treatment of patients with locally advanced or aggressive metastatic BC [37].

### 3.9. Fulvestrant

Fulvestrant (Figure 2) is a synthetic estrogen receptor and an aromatase inhibitor. It decreases the amount of estrogen in the BC cells through binding to estrogen receptors which leads to estrogen receptor deformation [38].

### 3.10. Lapatinib Oral-Active

Lapatinib (Figure 2), a synthetic quinazoline derivative, is an orally active reversible ErbB1 and ErbB2 tyrosine kinase receptor inhibitor that possesses antineoplastic activity towards breast cancer [39].

### 3.11. Pertuzumab

Pertuzumab is a recombinant humanized monoclonal antibody that targets HER2-positive BC which can inhibit the proliferation of human tumor cells [30]. The most common side effects of Pertuzumab include hair loss, low white blood cell count, rash, diarrhea, fatigue, nausea, and peripheral neuropathy (tingling in hands and feet and numbness) [40].

### 3.12. Alpelisib

Alpelisib (Figure 2) is a phosphatidylinositol 3-kinase (PI3K) inhibitor that impedes the growth of tumor cells. Alpelisib is used in combination with fulvestrant (Faslodex^®^) for treatment of postmenopausal women with a certain type of metastatic BC [41].

### 3.13. Talazoparib

Talazoparib (Talzenna^®^, Figure 2) belongs to a class of drugs called PARP inhibitors used for treatment of local advanced or metastatic HER2-negative BC women with a BRCA1 or BRCA2 mutation [42].

## 4. Recently Developed Synthetic Anti-Breast Cancer Small Molecules

### 4.1. Quinoline-Based Small Molecules

**Figure 3:**
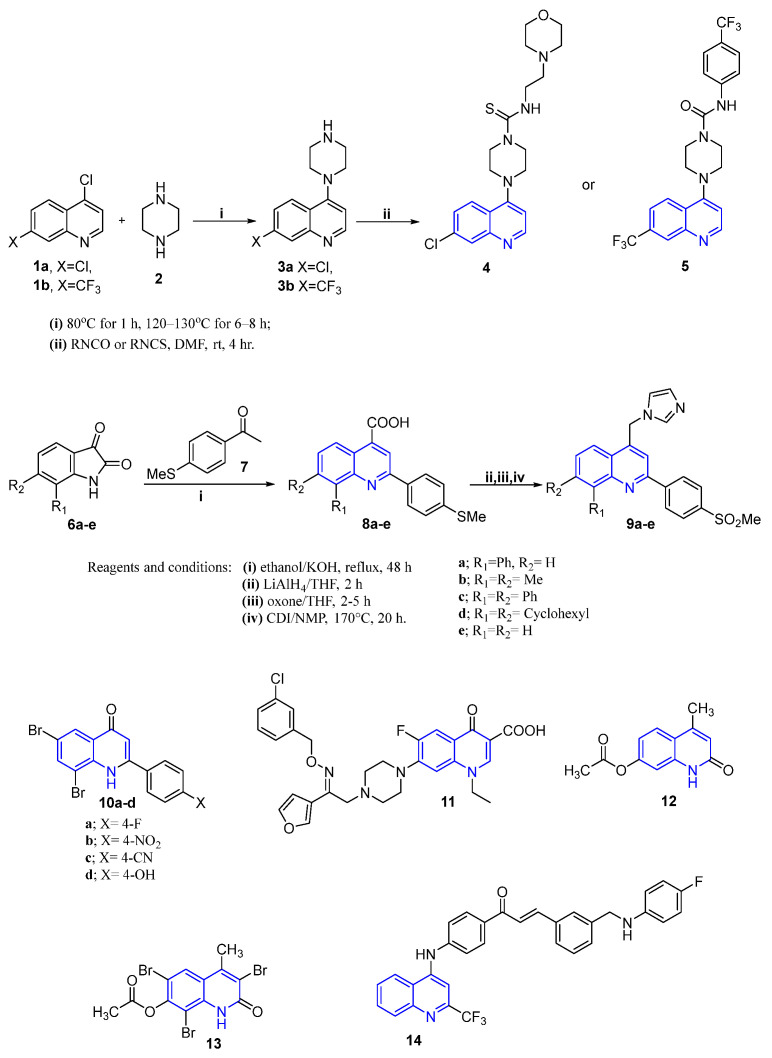
Quinoline-based small molecules as anti-breast cancer agents.

The great advances in the azaheterocyclic synthesis in the last decades allowed the synthesis of different quinoline derivatives. Recently, Viswas et al. reported the synthesis of two new sets of piperazinyl-quinoline derivatives bearing urea or thiourea functionalities based on the reaction of 4-(piperazin-1-yl)quinoline **3a**–**b** with isocyanate or thiocyanate derivatives. The synthesized quinolines were screened for their anti-proliferative activity against BC cell lines. The results highlighted that quinoline derivative **4** (Figure 3) possessed potent activity against MDA-MB-231 (GI_50_ = 3.0 ± 0.1 μM), while quinoline derivative **5** (Figure 3) exhibited improved anti-proliferative impact towards MDA-MB468 and MCF-7 (GI_50_ = 2.7 ± 0.1 and 2.0 ± 0.1μM, respectively) [43].

Previously, Zaghri et al. developed novel 4-(imidazolylmethyl)quinoline derivatives **9a**–**e** (Figure 3) that proved to be useful as anti-BC agents against MCF-7 cells; moreover, they exhibited selective cyclooxygenase-2 (COX-2) inhibitory activity. Among the aforementioned compounds, the results revealed that compound **9e** emerged as the most effective counterpart [44], (Figure 3).

Moreover, Bheemanapalli et al. [45] described the synthesis and anticancer activity evaluation of a new set of dihydroquinoline derivatives **10** and **11** (Figure 3). Preliminary screening revealed that compounds **10a**–**d** demonstrated potent growth inhibitory potential against breast cancer MCF-7 cell line compared to the reference drug. In addition, compound **11** (Figure 3) showed a significant growth inhibitory potential against three breast cancer cell lines: T47D, MCF-7 and MDA-MB-231, with IC_50_ values equal 2.20 ± 1.5, 3.03 ± 1.5 and 11.90 ± 2.6 μM, respectively [46].

Moustafa et al. [47] synthesized a novel series of 2-quinolinone derivatives and evaluated their anti-proliferative activity against the BC MCF-7 cell line. The results revealed that compounds **12** and **13** (Figure 3) elicited the highest anti-proliferative activity. Moreover, these compounds cause induction of apoptosis at pre-G1 phase, beside cell cycle arrest (G2/M) phases.

Most recently, Patel et al. [48] identified a novel class of 3-phenyltrifluoromethyl quinoline derivatives (Figure 3) and determined their anti-proliferative potential toward the breast cancer MCF-7 cell line. Among the synthesized derivatives, compound **14** displayed the highest anticancer activity with growth inhibition value in the nanomolar range (GI_50_ = 4 nM). The performed molecular docking analysis in this study suggests thymidine phosphorylase as a plausible target for the prepared 3-phenyltrifluoromethyl quinolines [48].

### 4.2. Quinazoline- and Quinazolinone-Based Small Molecules

**Figure 4:**
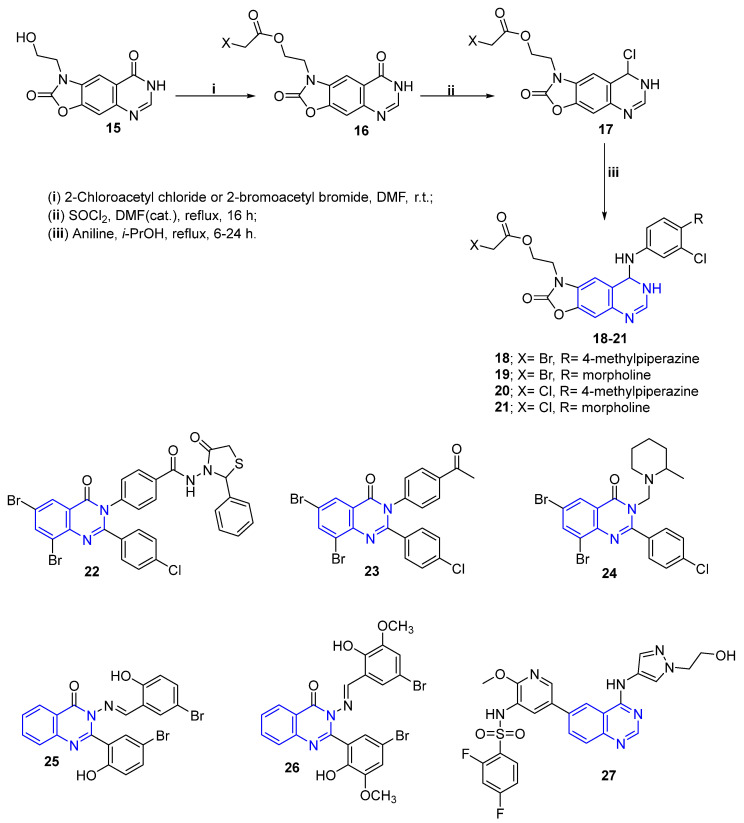
Quinazoline/quinazolinone derivatives as anti-breast cancer agents.

Several quinazoline and quinazolinone derivatives have been synthesized and reported for their anti-breast cancer activity. In 2016, Yin et al. reported the design and synthesis of a novel set of oxazolo-quinazoline derivatives (Figure 4) as dual inhibitors of EGFR/HER2. Among the synthesized compounds, **18**–**21** revealed significant inhibition for EGFR and HER2. Furthermore, compound **21** showed excellent anti-proliferation activity against the SKBr-3 cell line with IC_50_ = 0.47 ± 0.35 μM, compared to the reference drug Lapatinib (Table 1, Figure 4) [49].

Additionally, Ahmed et al. [50] discovered a new series of quinazolin-4-one derivatives and screened them against EGFR tyrosine kinase, as well as against the MCF-7 cell line. Compound **22** (Figure 4) revealed good EGFR inhibitory activity and powerful cytotoxic activity toward the tested MCF-7 cell line.

Furthermore, another novel series of quinazolin-4(3*H*)-one derivatives were designed, prepared and evaluated as anti-breast cancer agents [51], where compounds **23** and **24** (Figure 4) showed the highest activity against the MCF-7 cell line. Moreover, two new synthetic ellipticine analogs **25** and **26** (Figure 4) were designed and synthesized. Both compounds showed an elegant anti-proliferative effect for the MCF-7 cell line, with IC_50_ equal to 6.246 μmol/L and 5.910 μmol/L, respectively. Additionally, the two molecules were proven to induce intrinsic and extrinsic apoptosis at the cellular level [52].

Recently, Wang et al. developed a new 4-aminoquinazoline derivative **27** (Figure 4), and verified that it suppressed the proliferation, growth, migration, and invasion of human breast cancer cells. This was proven to occur via inhibiting the signaling pathway of PI3K/AKT/mTOR in vitro and in vivo with considerable safety profile [53].

### 4.3. Pyridine and Fused Pyridine Derivatives

**Figure 5:**
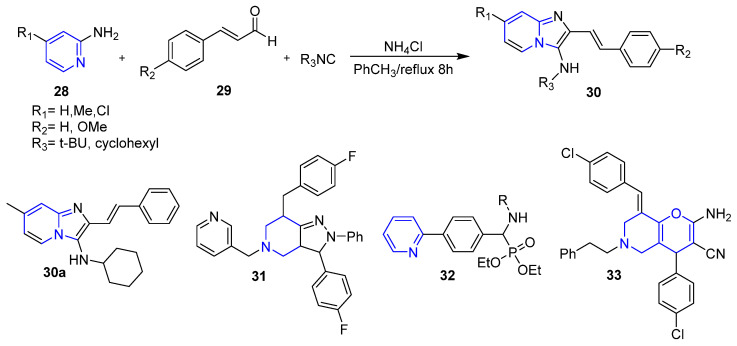
Certain reported pyridine and fused pyridine compounds as anti-breast cancer agents.

Most recently, Khalili et al. reported the design and synthesis of a new series of styrylimidazo[1,2-*a*]pyridine derivatives **30** (Figure 5) via reaction of cinnamaldehydes, 2-aminopyridines, and cyclohexyl/tert-butyl isocyanide mixture (Bienayme reaction). The synthesized compounds were screened against three breast cancer cell lines: MDA-MB-231, MCF-7, and T-47D using MTT assay. Most of the tested compounds displayed higher activity compared to the reference drug, etoposide. Fortunately, compound **30a** (Figure 5) showed the highest activity against the three examined breast cancer cell lines with IC_50_ values of 12.12 ± 0.54 µM, 9.59 ± 0.7 µM and 10.10 ± 0.4 µM, respectively. It is worth mentioning that the cytotoxic activity of the prepared styrylimidazo[1,2-*a*]pyridine derivatives is attributable to their ability to provoke apoptosis in the examined BC cell lines [54].

Moreover, Pang et al. prepared a novel series of pyrazolo[4,3-*c*]hexahydropyridine derivatives and studied their anti-proliferative activity against two breast cancer cell lines, MDA-MB-231 and MCF-7. The results showed that compound **31** (Figure 5) displayed excellent cytotoxic activity against MDA-MB-231 and MCF-7 with IC_50_ values of 4.2 µM and 2.4 µM, respectively, compared to standard drug 5-fluorouracil that showed IC_50_ values of 9.6 µM and 4.8 µM, respectively [55]. Further mechanistic insights, via cell cycle analysis, acridine orange/ethidium bromide (A.O./Et.Br.) double staining, and TUNEL assay, suggested that the target pyrazolo[4,3-*c*]hexahydropyridine derivatives could promote apoptosis in the tested BC cell lines.

Furthermore, Prasad et al. developed novel pyridine-bearing phosphonate esters **32** (Figure 5) as potential aromatase inhibitors. The design of these derivatives was performed utilizing computational docking analysis studies which revealed their ability to form strong H-bonds with the essential amino acid residues in the enzyme active site with considerable low energy. Accordingly, this serves as the mechanistic explanation for the significant in vitro cytotoxic activity observed by the compounds against the MCF-7 breast cancer cell line [56].

As a continuous search for novel potent anti-breast cancer agents, Rahnamay et al. reported the design and synthesis of a novel series of pyranopyridine derivatives. The anti-proliferative screening of the synthesized compounds identified compound **33** (Figure 5) as an effective cytotoxic agent against the MCF-7 cell line, with an IC_50_ value of 60 µM. Further cell cycle analysis, A.O./Et.Br. double staining and DNA fragmentation assay proved that compound **33** exerted its anti-proliferative activity by apoptosis induction in MCF-7 cells [57].

### 4.4. Pyridazine-Based Small Molecules

**Figure 6:**
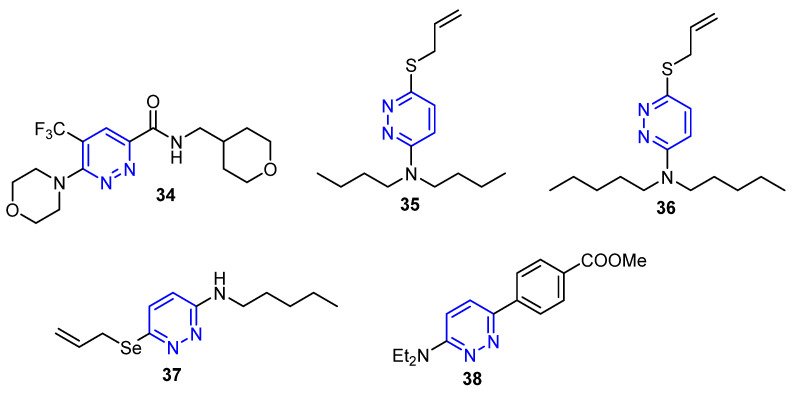
Pyridazine derivatives as anti-breast cancer agents.

In 2020, Sabt et al. designed and synthesized a new series of 3,6-disubstituted pyridazines (Figure 6) as promising antitumor agents targeting cyclin-dependent kinase 2 (CDK-2). Furthermore, the target 3,6-disubstituted pyridazines were in vitro evaluated for their growth inhibitory activity against three human cancer cell lines. The results showed that the compounds displayed selective cytotoxic activity against breast cancer cell lines, MDA-MB-231 and T-47D, while they revealed weak activity against ovarian cancer cell line SKOV-3. Among all these derivatives, compound **34** (Figure 6) was grasped as a potent cytotoxic agent against MDA-MB-231 and T-47D, with IC_50_ values of 0.99 ± 0.03 and 0.43 ± 0.01 μM, respectively [58].

Previously, Kim et al. developed a new series of 3-alkylamino-6-allylthio-pyridazine derivatives by using 3,6-dichloropyridazine as starting material and were evaluated in cytotoxicity assays. Among these, compounds **35** and **36** (Figure 6) revealed higher potencies against the MCF-7 cell line compared to the standard drug 5FU with IC_50_ values of 17.2 µg/mL, 17.16 µg/mL and 477.47 µg/mL, respectively [59]. Moreover, the authors prepared another series from 3-allylseleno-6-alkylthiopyridazines to investigate their biological activity as anti-breast cancer agents. For the derivatives tested, 3-allylseleno-6-pentylthiopyridazine **37** (Figure 6) exhibited the highest cytotoxic activity compared to 5FU against the MCF-7 cell line [60]. On the other hand, a new series of arylpyridazines was designed, synthesized and studied as anti-proliferative agents against seven cancer cell lines: HuH7, CaCo-2, MDA-MB-231, HCT116, PC3, NCI-H727 and HaCaT. Compound **38** (Figure 6) displayed favorable potent activity against all the tested cell lines with an IC_50_ value of 0.1 μM. Further mechanistic investigations revealed the ability of the target pyridazine derivatives to affect p44/42 and Akt-dependent signaling pathways [61].

### 4.5. Non-Fused and Fused Pyrimidines Moiety

**Figure 7:**
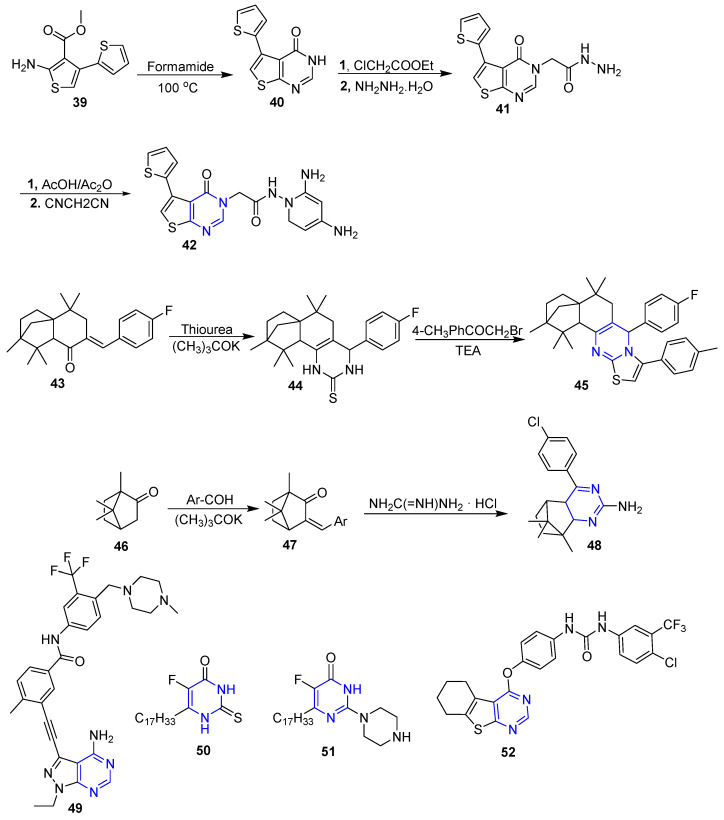
Non-fused and fused pyrimidines-based small molecules as anti-breast cancer agents.

Surveying the literature revealed that the pyrimidine entity serves as a promising scaffold for designing potent anti-breast cancer agents [62]. Accordingly, new derivatives of thieno[2,3-*d*]pyrimidine were synthesized and screened against MCF-7 breast cancer cell line. The results displayed that compound **42** (Figure 7) showed the most cytotoxic activity against breast cancer cell line MCF-7 with low IC_50_ with significant safety margin against non tumerogenic MCF-10A cell line. Further in vitro studies disclosed that the aforementioned compound exerts its cytotoxic activity through inhibition of pim-1 kinase [63].

In 2019, Wang et al. reported the design and synthesis of a series of isolongifoleno pyrimidine derivatives through three steps using fragment-based design approach. The anti-proliferative effect against breast cancer MCF-7 cell was estimated. Among all the tested pyrimidine derivatives, compound **45** (Figure 7) bearing 4-fluorophenyl and 4-methylphenyl moieties displayed maximum cytotoxic activity with IC_50_ value of 0.33 + 0.24 µM. Moreover, compound **45** was proven to induce apoptosis in the aforementioned cancer cells by boosting ROS generation [64].

Moreover, new compounds of camphor-based pyrimidine derivatives were synthesized and studied for their anticancer activity against three cancer cell lines: MDA-MB-231, A549 and RPMI-8226. According to the results of cytotoxicity assays, compound **48** (Figure 7) possessed the most potent cytotoxic activity against the tested cancer cell lines compared to the reference drug etoposide with much lower cytotoxic effect against normal cell GES-1 (IC_50_ > 50 µM vs. 8.89 µM). Noteworthy, the cytotoxic activity of compound **48** was attributable to the ROS-mediated mitochondrial apoptosis [65].

In addition, Zhang et al. reported the synthesis of 3-(phenylethynyl)-1*H*-pyrazolo[3,4-*d*]pyrimidin-4-amine derivatives (Figure 7) and the evaluation of their anti-proliferative activity towards the TNBC MDA-MB-231 cell line. Among the tested compounds, compound **49** (Figure 7) exhibited significant cytotoxic activity against the MDA-MB-231 TNBC cancer cell line. Moreover, it proved to be a potent multikinase inhibitor against Src with an IC_50_ value in the nanomolar range (0.9 nM), and against MAPK signaling protein kinases including B-RAF and C-RAF. Compound **49** also was proven to provoke apoptosis in the examined cancer cell line [66].

A series of pyrimidine *N*- and *S*-glycosides incorporating an oleyl residue were synthesized and evaluated against two cancer cell lines (MCF-7 and HepG2). The assay results outputs revealed that the tested compounds displayed moderate to high activities. Moreover, compounds **50** and **51** (Figure 7) exhibited the most potent activity against MCF-7 and HepG2 cancer cell lines with IC_50_ value of 13.2 µM and 24.9 µM, and 22.6 µM and 16.2 µM, respectively [67].

Moreover, a novel thienopyrimidine derivative (**52**) (Figure 7) was designed and synthesized as a VEGFR-2 tyrosine kinase inhibitor, a potential activity target of breast cancer. Thienopyrimidine **52** showed potent activity against T47D (IC_50_ = 6.9 ± 0.04 µM) and MDA-MB-231 (IC_50_ = 10 ± 0.04 µM). Additionally, compound **52** significantly inhibited VEGFR-2 by a percentage of 65%, and it down-regulated the level of VEGF in the MCF-7 cancer cell line by 30.4% which explained the molecular basis of the observed anti-proliferative activity [68].

### 4.6. Imidazole and Benzimidazole Derivatives

**Figure 8:**
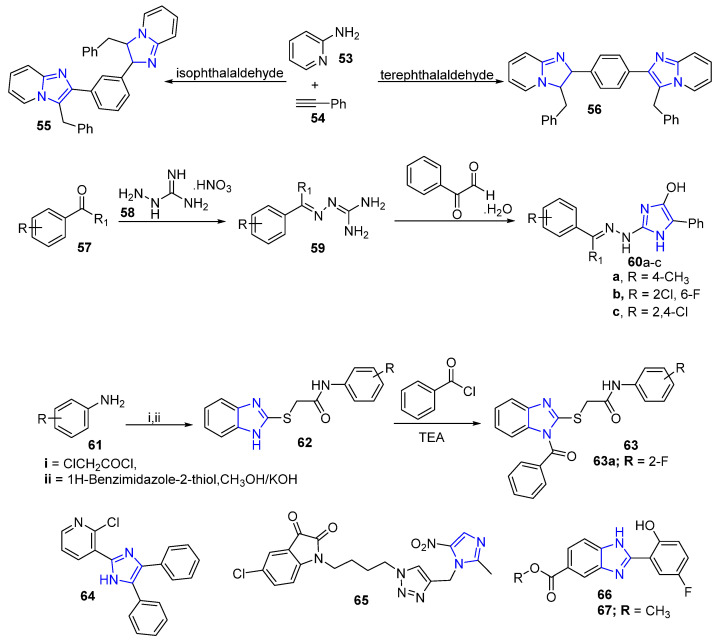
Imidazole and benzimidazole compounds as anti-breast cancer agents.

Meenakshisundaram et al. synthesized a new series of imidazoles and imidazopyridines via Schiff base reaction with a possibility of potential anticancer activity. The target compounds were evaluated for antitumor activities. Among these, compounds **55** and **56** were the most active against the three tested cancer cell lines: MDA-MB-231, HeLa and ACHN. Selectively, both compounds **55** and **56** (Figure 8) displayed effective significant activity against the breast cancer cell line MDA-MB-231 (**55**, GI_50_  =  0.30 µM; **56**, GI_50_  =  0.43 µM) [69].

The imidazole derivatives **60a**–**c** (Figure 8) were synthesized through a condensation reaction between phenylglyoxal monohydrate with guanidnyl hydrazone. All target imidazoles were screened for human breast cancer cell line MCF-7 by using the MTT assay. Results showed that compounds **60a**–**b** (Figure 8) revealed higher cytotoxic activities compared to the reference drug (5-FU and irinocam) against the aforementioned cancer cell line giving insight that this scaffold can serve as a good platform for designing novel anti-breast cancer agents [70]. Moreover, new benzoimidazole derivatives **63** (Figure 8) were designed and synthesized to explore their anti-proliferative activity. In particular, compound **63a** (Figure 8) exhibited the highest anticancer activity (IC_50_ = 0.0047 µM/mL) against the MCF-7 cancer cell line with almost equal cytotoxicity compared to the reference drug tamoxifen [71].

Mohan et al. [72] synthesized new compounds based on imidazole scaffold as anti-BC agents through inhibition of the signaling pathway of PI3K/Akt/mTOR. Anticancer results revealed that compound **64** (Figure 8) demonstrated potent activity against two breast cancer cell lines, MDA-MB-231 and MCF-7. Additionally, a novel series of imidazole–isatin–thiosemicarbazone and 1,2,3-triazole tethered imidazole–isatin hybrids were designed, synthesized and studied as anti-breast cancer agents (Figure 8). It was found that compound **65** was the most potent counterpart with the value of IC_50_ 26.12 μM and 54.25 μM against MDA-MB-231 and MCF-7 cell lines, respectively [73].

Karthikeyan et al. [74] synthesized a new series of benzimidazole carboxylic acids and their esters **66** (Figure 8) as novel potential anti-BC agents. In particular, ester **67** (Figure 8) possessed the strongest anti-proliferative activity against three breast cancer cell lines, MCF-7, MDA-MB-231 and MDA-MB-468, with the value of growth inhibition GI_50_ = 0.18, 4.09 μM and 6.23 μM, respectively.

### 4.7. Coumarin Derivatives

**Figure 9:**
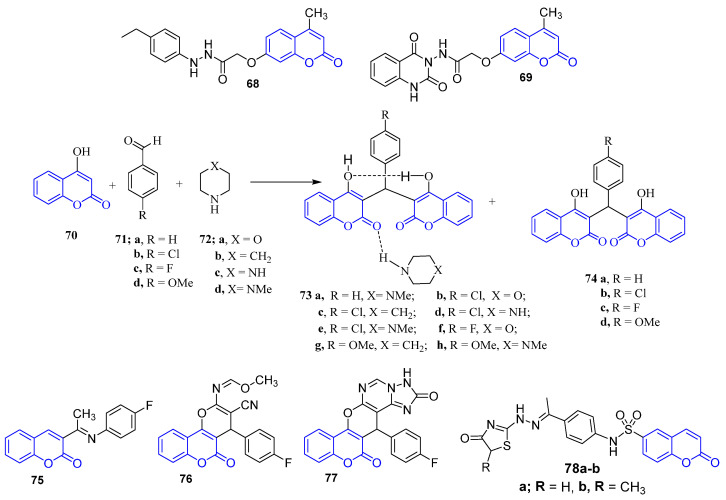
Coumarin-based small molecules as anti-breast cancer agents.

Ahmed et al. designed and synthesized twenty-five coumarin-based derivatives and evaluated their anti-proliferative activity against the MCF-7 cancer cell line and their VEGFR-2 kinase inhibitory activity. Compounds **68** and **69** (Figure 9) displayed a maximal significant response against the MCF-7 cancer cell line with an IC_50_ value of 1.24 µM and 1.65 µM, respectively, comparable to that of the reference drug staurosporine (IC_50_ = 8.81 µM). Furthermore, compound **68** was able to inhibit VEGFR-2 kinase activity at an IC_50_ of 0.36 µM which is comparable for that recorded by staurosporine (IC_50_ = 0.33 µM) [75].

The condensation reactions of 4-hydroxycoumarin, aldehydes and cyclic secondary amines yielded the bis-coumarins derivatives **73** and **74** (Figure 9). A cytotoxicity of the novel coumarin derivatives against the MCF-7 breast cancer cell line was evaluated and displayed that compound **73** has a promising cytotoxic activity against MCF-7 with an IC_50_ value of 12.1 µg/mL which is superior to that of the reference drug doxorubicin with an IC_50_ value of 16.2 µg/mL. A molecular docking study for the target compounds identified Topoisomerase IIa as a potential target [76].

New compounds of fluorinated coumarin-based derivatives were synthesized, and their anti-proliferative activity was studied on MCF-7 and HeLa in addition to their potential to inhibit VEGFR-2. Compounds **75**, **76** and **77** (Figure 9) exhibited higher inhibition activity against the MCF-7 cell line (IC_50_ = 8.30 μg/mL, 8.28 μg/mL, and 7.90 μg/mL, respectively). Furthermore, these coumarins unveiled superior inhibitory activity against VEGFR-2 with inhibition percentage of 94% [77]. In 2018, Sabt et al. developed new series of coumarin sulfonamide derivatives and evaluated their anticancer activity. Among all these derivatives, compounds **78a**–**b** (Figure 9) (IC_50_ =10.95 ± 0.96 µM and IC_50_ = 10.62 ± 1.35 µM) exhibited significant cytotoxic activity against breast cancer MCF-7 cell lines [78]. Interestingly, the target coumarin-6-sulfonamides efficiently promote the mitochondrial apoptosis.

### 4.8. Tetrazole-Bearing Derivatives

**Figure 10:**
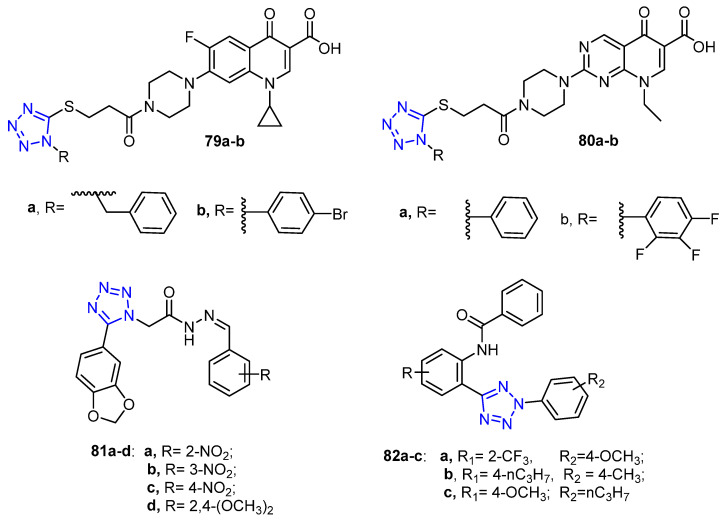
Tetrazole-bearing compounds as anti-breast cancer agents.

Dileep and coworker described a novel series of tetrazole-bearing ciprofloxacin and pipemidic acid derivatives and screened their anti-proliferative activity against the MDA-MB-231 breast cancer cell line. Based on the results assay, compounds **79a**–**b** and **80a**–**b** (Figure 10) displayed the most potent cytotoxic activity with growth inhibition ranging (GI_50_ = 0.08–0.09 µM), which is significantly more potent than the reference drug tamoxifen (GI_50_ = 0.24 µM) against the MDA-MB-231 cell line [79].

In another study, Arshad and coworkers prepared a series of tetrazole-based derivatives and evaluated the biological activity of these compounds against MCF-7 (ER positive), MDA-MB-231 and ZR-75 (ER negative) breast cancer cell lines. The results showed that compounds **81a**–**c** (Figure 10) exhibited potent inhibitory activity against MCF-7 cells, while compound **81d** (Figure 10) revealed potent activity against both ZR-75 and MDA-MB-231 cell lines [70]. Furthermore, compounds **82a**–**c** unveiled higher potent selectivity toward breast cancer resistance protein (BCRP/ABCG2) than the reference Ko143 (Figure 10) [80].

### 4.9. Indole- and Oxindole-Based Anti-Breast Cancer Agents

**Figure 11:**
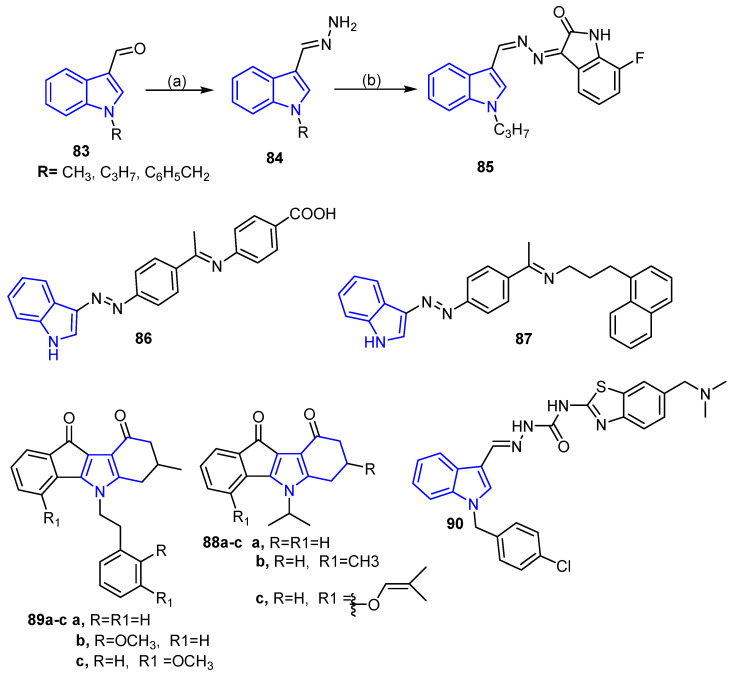
Indole and oxidole compounds as anti-breast cancer agents.

Indole and oxindole moiety, among the widest-spread heterocycles in nature, are used as a building block for many pharmaceutical agents, especially in the discovery of new antitumor agents [81,82,83,84]. For example, Eldehna et al. in 2018 [85] developed a new series of [(3-indolylmethylene) hydrazono]indolin-2-ones derivatives and investigated their cytotoxic activity. Compound **85** (Figure 11) was the most active derivative against the MCF-7 cell line with an IC_50_ value of 1.04 ± 0.08 μM which is higher than the reference drug doxorubicin (IC_50_ = 2.57 ± 0.18 μM) and was capable of inducing apoptosis and cell cycle arrest at the G2/M phase. Moreover, Kaur et al. developed a new series of indole hybridized diazenyl derivatives. Among the tested derivatives, compounds **86** and **87** (Figure 11) possessed promising activity against breast cancer cell line MDA-MB-231, in addition to being safe by exhibiting very low cytotoxic activity against the normal cell line [86].

Novel indole derivatives were also designed and synthesized to investigate their biological activity as casein kinase II (CK2) and ABCG2 inhibitors. The results revealed that the compounds having N^5^-isopropyl substituent on the C-ring (**88a**–**c**) were the most potent inhibitors of casein kinase II (CK2) (IC_50_ = 0.17–0.36 μM) (Figure 11), while compounds containing N^5^-phenethyl substituent on the C-ring (**89a**–**c)** displayed significant activity against breast cancer resistance protein ABCG2 (IC_50_ = 0.21–0.31 μM) (Figure 11) [87].

In 2015, Ma et al. synthesized a novel series of indole-benzothiazole derivatives and examined their antitumor activity against four cancer cell lines: HT29, H460, A549 and MDA-MB-231. The assay results showed that compound **90** (Figure 11) displayed potent cytotoxic activity against MDA-MB-231 with IC_50_ values of 0.024, 0.29, 0.84 and 0.88 μM, respectively; this was explained by their ability to activate procaspase-3, besides arresting the cell cycle [88].

**Figure 12:**
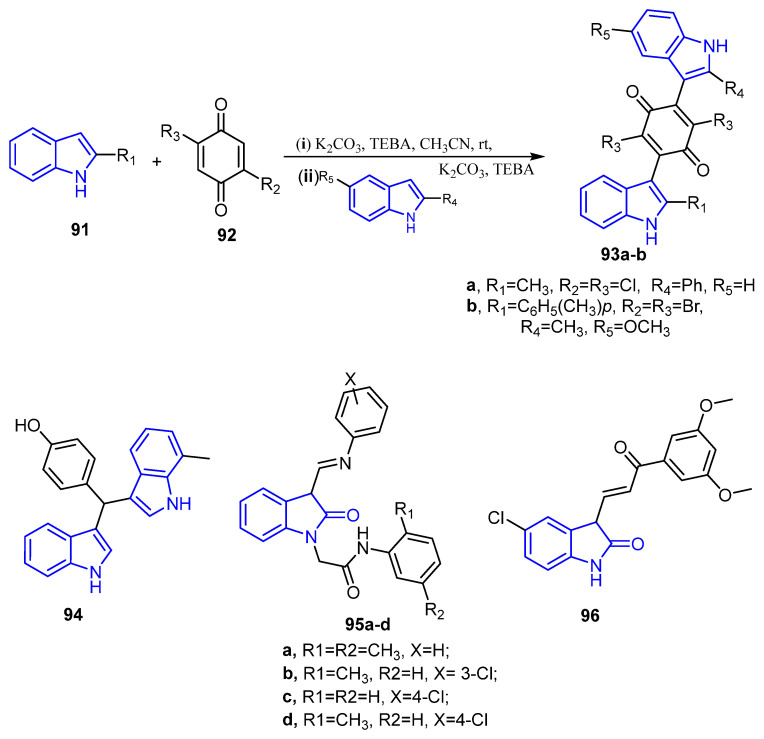
Indole and oxidole compounds as anti-breast cancer agents.

The reaction of 2-substituted indoles with halogeno-quinone produced indolylquinone derivatives which were designed as potential anti-breast cancer agents. The results exhibited that compound **93a** displayed significant potency toward MCF-7 (IC_50_ = 2.29 μg/mL), while compound **93b** was the most active derivative against MDA-MB-231 (IC_50_ =3.99 μg/mL) (Figure 12) [89]. Fluorescence microscopy analysis hinted that indolylquinone derivatives inhibited the growth of BC via triggering apoptotic cell death.

Furthermore, aryl methyl ring substituted analog of 3,3′di Indolyl methane (DIM), Phemindole **94** (Figure 12), was synthesized and evaluated as an anticancer agent toward triple-negative breast cancer (TNBC). This compound exhibited potent activity against MDA-MB-231 cell lines with induction of apoptosis in MDA-MB-231 cells and have anti-migration activity by focal adhesion kinase control phosphorylation in the aforementioned cell line [90].

In 2016, Debnath et al. synthesized ten new oxindole analogs. Cytotoxicity evaluation revealed that especially compounds **95a**–**d** displayed potent activity against MCF-7 (Figure 12) with the value of growth inhibition (GI_50_ < 0.02 µM) comparative to activity with reference drug adramycin (doxorubicin). A molecular docking into the EGFR biding site (PDB ID 1M17) has highlighted EGFR kinase as a possible target for the oxindole derivatives [91].

Karthikeyan et al. developed new indolin-2-ones derivatives bearing oxindole and chalcones moieties as anti-proliferative and breast cancer agents. All of the synthesized compounds exhibited promising anticancer activity against the tested cell lines. In particular, 5-chloro-3-(2-(3,4-dimethoxyphenyl)-2-oxoethylidene) indolin-2-one (**96**) with GI_50_ = 3.59, 4.76 and 8.54 µM (Figure 12) showed potent cytotoxic activity toward the three tested breast cancer cell lines, MCF-7, MDA-MB-468 and MDA-MB-231, respectively [92].

### 4.10. Triazine-Based Derivatives as Anti-Breast Cancer Agents

**Figure 13:**
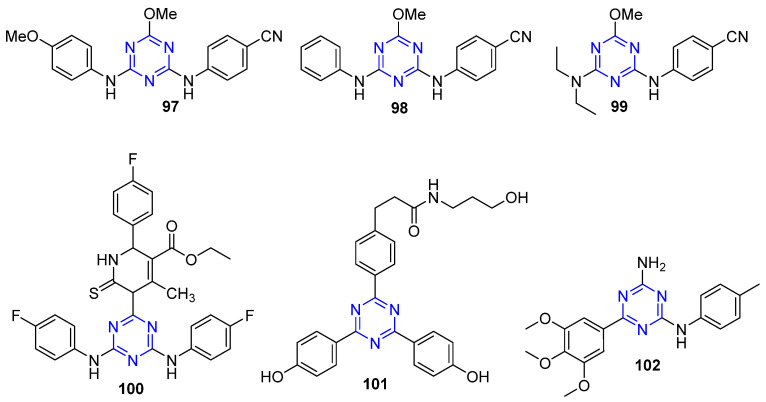
Triazine compounds as anti-breast cancer agents.

El-Faham et al. developed novel derivatives of di- and tri-substituted *s*-triazine as potential target of MCF-7 (ER+) and MDA-MB-231 (ER−) human BC cell lines. Compounds **97** and **98** (Figure 13) displayed potent anticancer activity against the MCF-7 cancer cell line with IC_50_ values less than 1 µM (0.77 ± 0.01 and 0.1 ± 0.01 μM, respectively). Furthermore, compound **99** (Figure 13) exhibited the better cytotoxic activity against MDA-MB-231 with an IC_50_ value of 6.49 ± 0.04 µM [93]. The study outcomes suggested that target triazines exerted their anticancer action in human BC cells through targeting the estrogen and progesterone receptors.

Moreover, Srivastava et al. utilized a three-component reaction using Bi(NO_3_)_3_ as a catalyst in one-pot synthesis approach to prepare some hybridized analogues of monastrol-1,3,5-triazine. The synthesized compounds exhibited anti-proliferative activity selectively against the MCF-7 breast cancer cell line where the most active compound **100** (Figure 13) displayed an IC_50_ value of 41.5 + 0.31 µM. Moreover, all the tested compounds proved to be non-toxic to the normal cell line MCF-12A. Interestingly, the observed anti-proliferative activity was justified by the marked inhibition of EGFR tyrosine kinase tested in vitro and in vivo by all the synthesized compounds. Fortunately, compound **100** was able to inhibit the target enzyme by 96.4% [94].

In 2020, Hu et al. published interesting research where they designed and synthesized novel 1,3,5-triazine ring bearing acrylic acid or acrylic amide side chains as selective estrogen receptor degraders (SERDs) besides their possessing estrogen receptor antagonism properties. Degrading estrogen receptors is a new and effective approach for combating breast cancer. Unfortunately, only few scaffolds proved to be beneficial as SERDs. Through their research, Hu et al. proved that all the target compounds possess marked anti-BC activity. Compound **101** (XHA1614) (Figure 13) exhibited potent remarkable cytotoxicity versus Ishikawa and MCF-7 cells (IC_50_ = 3.11 μM and 3.15 μM, respectively) and also led to significantly degrading ER level at 1 nM in Western blotting assay, beside its antagonistic activity against progesterone receptor in MCF-7 cells. Accordingly, the triazine scaffold proved to be a promising candidate as SERD for the management of breast cancer [95]. Moreover, in the same year, Junaid et al. prepared a novel series of 6, *N*^2^-diaryl-1,3,5-triazine-2,4-diamine derivatives to screen the cytotoxic activity against three breast cancer cell lines: MDA-MB-231, MCF-7 and SKBR-3. The results indicated that the synthesized compounds have a significant activity against MDA-MB-231; in particular, compound **102** (Figure 13) exhibited excellent anti-proliferative activity with IC_50_ = 1 nM without cytotoxicity against the normal cell of MCF-10A breast [96].

### 4.11. Oxadiazole-Bearing Small Molecules as Anti-Breast Cancer Agents

**Figure 14:**
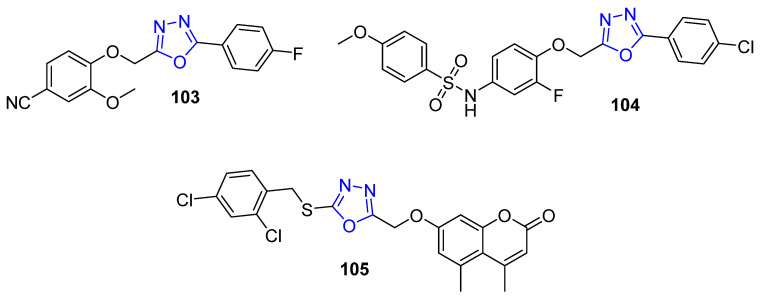
Oxadiazole compounds as anti-breast cancer agents.

A series of novel substituted 2-(phenoxymethyl)-5-phenyl-1,3,4-oxadiazole derivatives was prepared and screened for cytotoxicity against BC cells. The target compounds unveiled good activity towards the tested cell lines, in particular, compound **103** (Figure 14), the most potent derivatives with IC_50_ = 10.25 ± 2.5 and 10.51 ± 1.9 µM against BC MCF-7 and MDA-MB-453 cell lines, respectively. Moreover, the target 2-(phenoxymethyl)-5-phenyl-1,3,4-oxadiazoles revealed their ability to induce apoptosis [97].

Gamal El-Din et al. developed a series of 1,3,4-oxadiazole derivatives bearing a sulfonamide moiety in order to screen their anticancer activity against a panel of 58 cancer cell lines of nine different types of cancer. Compound **104** (Figure 14) showed a powerful cytotoxic effect against tested cell lines, especially the T-47D breast cancer cell line with growth inhibition of 90.47% at 10 µM, compared to the reference drugs Paclitaxel and Gefitinib [98]. Additionally, a novel series of coumarin linked to 1,3,4-oxadiazole moiety was synthesized and screened as anti-BC agents against two breast cancer cell lines, MCF-7 and MDA-MB-231. Compound **105** (Figure 14) displayed the most effective potency against MCF-7 cells with an IC_50_ value < 5 µM which is about 1.4 times more potent than the reference drug tamoxifen [99].

### 4.12. Thiazolidine Derivatives

**Figure 15:**
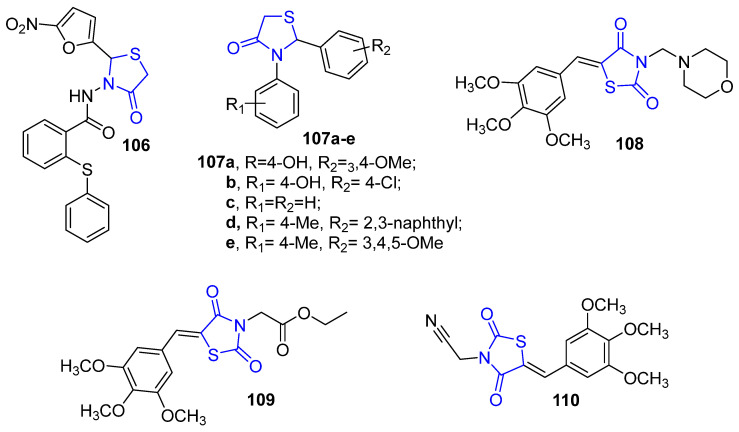
Thiazolidine derivatives as anti-breast cancer agents.

Recently, a new series of thiazolidinone derivatives was synthesized to investigate their bioactivity as anti-breast cancer agents. The results exhibited that compound **106** (Figure 15) revealed to be the most active compound with IC_50_ values of 1.9 ± 1.15, 5.4 ± 1.13 and 6.5 ± 1.16 μM against MDA-MB-231, HepG2 and HT-29, respectively. This compound unveiled a promising anticancer agent against TNBC which induces apoptosis via arresting cell cycle at G1/S phase, beside inhibition of angiogenesis [100]. In addition, a new family of 2,3-thiazolidin-4-one derivatives was reported as effective anti-BC agents. Compounds **107a**–**b** (Figure 15) showed potent cytotoxicity towards MCF-7 cells, while compounds **107c**–**e** (Figure 15) showed remarkable activity against SKBR3 cells [101].

El-Kashef et al. synthesized 3,5-disubstituted thiazolidine-2,4-dione derivatives and evaluated their anti-proliferative potential against MCF-7 and MDA-MB-231 cancer cell lines using MTT assay. The results displayed that compounds **108**, **109** and **110** (Figure 15) were the most potent against MCF-7 with the IC_50_ value of 1.27, 1.31 and 1.50 μM, respectively. Furthermore, these three compounds, **108**–**110**, induced apoptosis via reducing the expression levels of the anti-apoptotic protein Bcl-2 and enhancing the expression level of the pro-apoptotic protein Bcl-2 [102].

### 4.13. Anti-Breast Cancer Agents Incorporating Naphthalene, Isoxazole and Pyrazole Moieties

**Figure 16:**
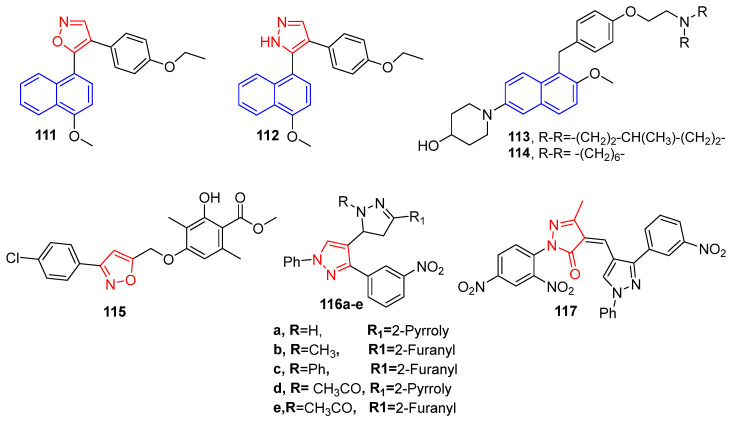
Naphthalene scaffold as anti-breast cancer agents.

Wang et al. reported a new series of isoxazole-naphthalene derivatives as tubulin polymerization inhibitors. Their anti-proliferative activity was evaluated, and according to the assay results, compound **111** (Figure 16) bearing 4-ethoxy substitution at phenyl ring (IC_50_ =1.23 ± 0.16 μM) was the most potent against MCF-7 cancer cell line. Moreover, this compound **111** (Figure 16) has the ability to repress tubulin polymerization with an IC_50_ value of 3.4 μM, beside apoptosis induction, and ultimately cell cycle arrest (G2/M) phase [103]. In addition, the same research group has reported on another series from pyrazole-naphthalene derivatives. Compound **112** (Figure 16) with IC_50_ = 2.78 ± 0.24 μM against MCF-7 cell line, was five times more potent than the reference drug cisplatin (IC_50_ = 15.24 ± 1.27 μM). Furthermore, the aforementioned compound **112** (Figure 16) inhibited tubulin polymerization with IC_50_ value of 4.6 μM in addition to induction of apoptosis and cell cycle arrest at the G2/M phase [104].

Interestingly, Jha et al. designed and synthesized a new series of 6-(4-hydroxypiperidino)naphthalen-2-ol derivatives as selective estrogen receptor modulators (SERMs). Among all the tested compounds against the MCF-7 cancer cell line, compounds **113** and **114** (Figure 16) exhibited the higher cytotoxicity to oestrogen-responsive human breast cancer MCF-7 compared to the standard drug tamoxifen. Moreover, compound **114** (Figure 16) showed significant binding and antagonistic effects against human ER in an in vitro assay [105].

A novel series of sixteen methyl β-orsellinate-based 3,5-disubstituted isoxazole derivatives was synthesized by Reddy et al. The target compounds were screened in vitro for their anticancer activity against four cancer cell lines: IMR-32, DU-145, MIAPACA and MCF-7. Compound **115** (Figure 16) showed the highest inhibitory potency against the MCF-7 cell line (IC_50_ = 7.9 ± 0.07 μM) with an induction of apoptosis and cell cycle arrest at the G2/M phase [106].

A series of new pyrazole derivatives has been synthesized and screened in vitro as anti-BC targeting VEGFR-2 kinase. The assay results displayed that compounds **116a**–**e** and **117** (Figure 16) exhibited the most potent activity against MCF-7 cell line with the value of IC_50_ ranging (16.50–26.73 μM) compared to tamoxifen (IC_50_ = 23.31 μM). Furthermore, the synthesized compounds, especially **116b**–**e** and **117**, showed significant inhibitory activities toward VEGFR-2 kinase with inhibition activity (70–79%). In particular, compounds **116c**, **116e** and **117** unveiled the most inhibitory efficiency with IC_50_ in nanomolar range (913.51, 225.17, and 828.23 nM, respectively) in comparison to the reference drug sorafenib (IC_50_ = 186.54 nM) [107].

### 4.14. Benzofuran Derivatives

**Figure 17:**
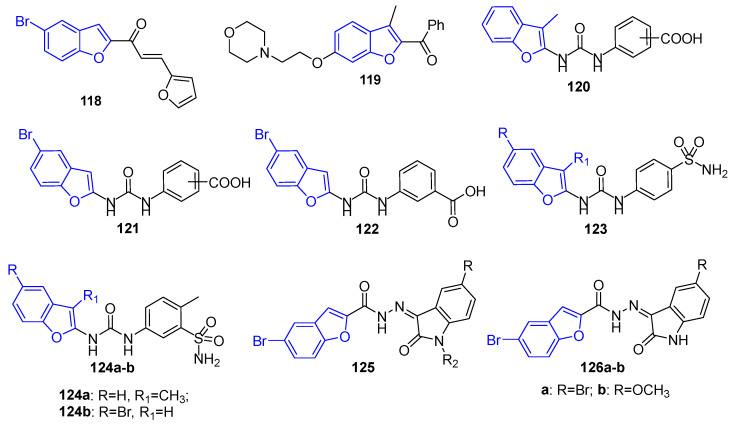
Benzofuran derivatives as anti-breast cancer agents.

Benzofuran-based small molecules are well known in medicinal chemistry for their diverse, broad spectrum anticancer activity. Coskun et al. designed and synthesized a new series of benzofuran substituted chalcone derivatives and studied their in vitro antitumor activities by MTT assay. The results indicated that the tested compounds revealed cytotoxic activity against malignant MCF-7 and PC-3 cell lines, in particular compound **118** (Figure 17) with the value of log IC_50_ = 0.42 and 0.67 μM, respectively [108].

It is also reported that a novel series of benzofuran derivatives was designed and synthesized as potential oestrogen receptor inhibitors endowed with anti-breast cancer activity. Among these compounds, 2-benzoyl-3-methyl-6-[2-(morpholin-4-yl)ethoxy] benzofuran (**119**) (Figure 17) owned the most potent activity against MCF-7 human breast cancer cells with inhibitory percentage of 64.23% at 50 µM and showed a low toxicity toward normal cells. Moreover, a comprehensive structure–activity relationship was extracted from this study [109].

In 2020, Eldehna et al. reported the design and synthesis for new 2-methylbenzofuran **120** or 5-bromobenzofuran **121** derivatives featuring the carboxylic acid functionality represented in the benzoic and hippuric acid moieties (Figure 17). The target 2-methylbenzofuran or 5-bromobenzofuran derivatives were assessed for their inhibitory action against cancer-related human carbonic anhydrases (*h*CA) IX and XII isoforms. They displayed good inhibitory activity and selectivity toward *h*CA IX isoform (*K*_I_s ranging from 0.56 to 5.1 µM), and subsequently the most potent inhibitors were screened for their potential antitumor impact against two human breast cancer cell lines, MCF-7 and MDA-MB-231. In particular, 5-bromobenzofuran-based counterpart **122** exerted the best anti-proliferative action against the examined TNBC MDA-MB-231 cells (IC_50_ = 2.52 ± 0.39 µM) which was comparable to doxorubicin (IC_50_ = 2.36 ± 0.18 μM). Moreover, compound **122** significantly elevated (from 0.78 to 31.88%) the Annexin V-FITC positive MDA-MB-231 apoptotic cells, as well as led to a cell cycle disturbance through alteration of Sub-G_1_ phase and arrest of G_2_-M stage (Figure 17) [110].

One year later, in 2021, novel sets of ureido benzofurans incorporating sulfonamide functionality (**123**, Figure 17) were developed as anti-breast cancer agents targeting cancer-related *h*CA IX and XII isoforms. Several derivatives potently inhibited the cancer-related *h*CA IX isoform within the single-digit nanomolar range (*K*_I_s: 1.8–8.4 nM). Further MTT assay was performed, which ascribed potent anti-proliferative impact to **124a** against BC MCF-7 cells (IC_50_ = 6.45 µM) and MDA-MB-231 cells (IC_50_ = 6.27 µM), whereas **124b** exhibited moderate activity toward BC MCF-7 cells (IC_50_ = 13.79 µM) and MDA-MB-231 cells (IC_50_ = 14.16 µM). Further investigations revealed that treatment of BC MCF-7 and MDA-MB-231 cells with ureido benzofuran **124a** led to up-regulation of the expression levels of pro-apoptotic Bax and Caspase-3 proteins, and down-regulation for anti-apoptotic Bcl-2 protein expression level (Figure 17) [111].

Additionally in 2021, the design and synthesis of novel sets of 5-bromobenzofuran-based small molecules tethered with indolin-2-one (**125**, Figure 17) moiety were described by Eldehna et al. [112]. The target benzofuran–indolinone conjugates were developed as dual inhibitors for the two key oncotargets CDK2/GSK-3β that are involved in breast cancer. All the reported benzofuran–indolinone conjugates in this study were screened for their anti-breast cancer activity towards T-47D and MCF-7 cell lines. Superiorly, conjugates incorporating *N^1^*-unsubstituted indolinone moieties exerted moderate to potent activity toward both T-47D (IC_50_: 1.27 ± 0.04–9.67 ± 0.31 µM) and MCF-7 (IC_50_: 2.27 ± 0.06–12.93 ± 0.38 µM) cell lines. Moreover, a CDK2 and GSK-3β enzyme inhibition assay, for conjugates incorporating *N*^1^-unsubstituted indolinone moieties, identified benzofuran-based conjugates **126a**–**b** (Figure 17) as potent dual CDK2/GSK-3β inhibitors with IC_50_ values equal to 37.77 and 52.75 nM, respectively, toward CDK2, and IC_50_ values equal to 32.09 and 40.13 nM, respectively, toward GSK-3β.

## 5. Miscellaneous Anti-Breast Cancer Agents

**Figure 18:**
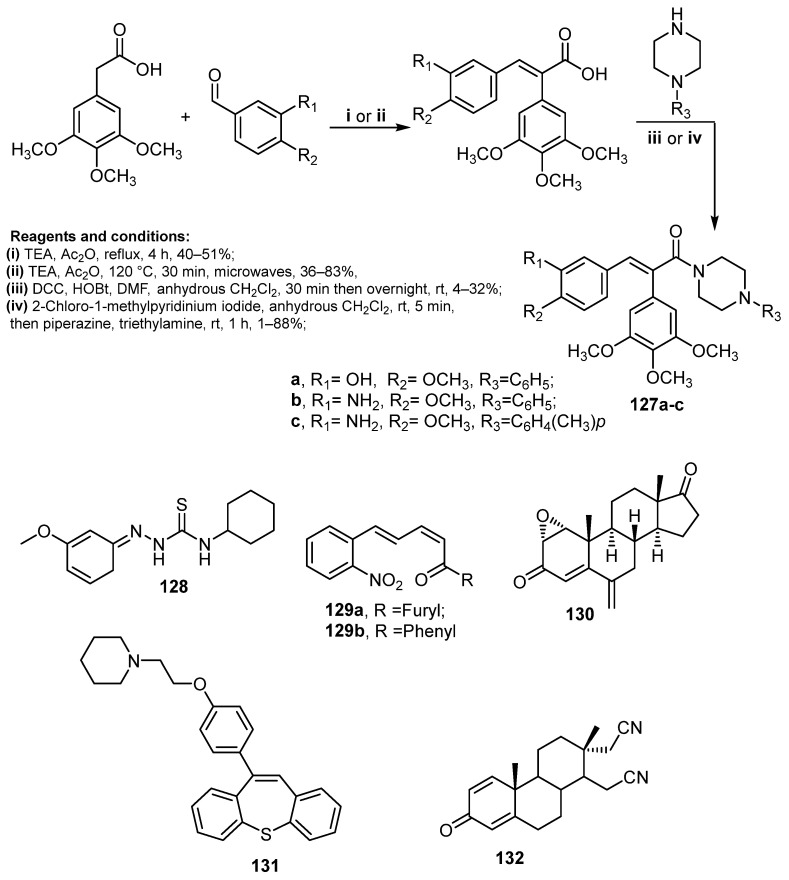
Miscellaneous compounds as anti-breast cancer agents.

A new series of thirty-three combretastatin A-4 (CA-4) analogs conjugated to piperazine moiety was synthesized via Perkin reaction by Boyle et al. in 2019 [113] and tested against MCF-7 breast cancer cells. Compounds **127a**–**c** (Figure 18) showed potent anti-proliferative activity against MCF-7 cells with a range of IC_50_: 83–190 nM, as well as induced apoptosis via arresting cell cycle G2/M phase in MCF-7 cancer cell line. Moreover, the developed CA-4 analogs were able to act directly on tubulin as microtubule-destabilizing agents. Moreover, another research group developed a novel series of cyclohexyl thiosemicarbazone derivatives to screen their cytotoxic activity. Compound **128** (Figure 18) displayed the highest activity against three breast cancer cell lines: MDA-MB-468, MDA-MB-231 and SKBr-3, with a range of IC_50_ values of 24.50 ± 0.01–32.2 ± 0.09 µM. Unfortunately, the aforementioned compounds did not show any activity against breast cancer MCF-7 cell lines [114].

In 2014, Weldon et al. synthesized a series of cinnamylidene acetophenones and studied their anticancer activity. The results indicated that compounds **129a**–**b** (Figure 18) displayed sub-micromolar activity toward MDA-MB-468 and MCF-7 cells with relatively less activity against normal cell MCF-10A cells [115]. In addition, Varela and coworkers synthesized three metabolites of the steroidal aromatase inhibitor exemestane and tested them against MCF-7aro cells. Steroid **130** (Figure 18) displayed the most potent inhibition of MCF-7aro cells viability with IC_50_ of 0.25 μM. In addition, it induces loss of plasma membrane integrity [116].

Ansari et al. [117] synthesized two series of dibenzo[b,f]thiepines and dibenzo[b,f]oxepines, and then tested their anticancer activity against breast cancer (MDA-MB-231 and MCF-7) cell lines. Assay results displayed that compound **131** (Figure 18) exhibited the highest activity with IC_50_ values of 1.33 µM for MCF-7 and 5 µM for MDA-MB-231 with cell cycle arrest at G0/G1 phase in MCF-7. Moreover, compound **132** (Figure 18) revealed excellent anti-proliferative activity against breast cancer (MDA-MB-231 and MCF-7) cell lines with IC_50_ values = 0.11 and 0.52 µM, respectively [118].

**Figure 19:**
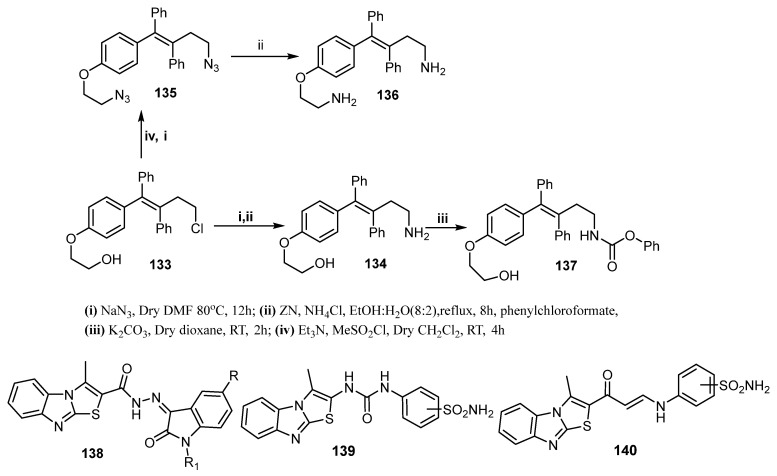
Miscellaneous compounds as anti-breast cancer agents.

Furthermore, Kaur et al. developed a novel series of ospemifene analogs and tested their cytotoxic activity against MDA-MB-231 (ER-negative) and MCF-7 (ER-positive) cancer cell lines. The novel compounds **134** and **136** (Figure 19) were found to be more effective than the reference (ospemifene and tamoxifen) against MDA-MB-231 cells (IC_50_ = 25 and 17.1 µM, respectively) and against MCF-7 cells (IC_50_ = 15.9 and 23.6 µM, respectively). Moreover, compound **137** (Figure 19) showed potent cytotoxic activity against MCF-7 cells (IC_50_ = 76 µM) comparable to that of ospemifene and tamoxifen [119].

Recently, Eldehna et al. reported two studies concerning development of novel thiazolo[3,2-*a*]benzimidazole derivatives as antitumor agents against breast cancer cell lines. In the first study, the thiazolo[3,2-*a*]benzimidazole moiety was conjugated with different isatin motifs (**138**, Figure 19) to afford new CDK2 inhibitors (IC_50_: 26.24 ± 1.4–96.46 ± 5.3 nM) with potent activity against BC MCF-7 cell line (IC_50_: 1.27 ± 0.06–16.83 ± 0.95 μM) and against MDA-MB-231 cell line (IC_50_: 2.60 ± 1.47–20.90 ± 1.17 μM) [120]. The second study reported the conjugation of the thiazolo[3,2-*a*]benzimidazole framework with a benzenesulfonamide moiety through urea (**139**, Figure 19) or enaminone (**140**, Figure 19) linkers to furnish a novel class of potent and selective inhibitors of cancer-related *h*CA IX and XII isoforms with inhibition constants in the nanomolar range. The most potent CA inhibitors displayed further efficient cell growth inhibitory activity against BC MCF-7 and MDA-MB-231 cell lines under both normoxic and hypoxic conditions. Further investigations revealed their impact on induction of apoptosis and cell cycle progression [121].

**Figure 20:**
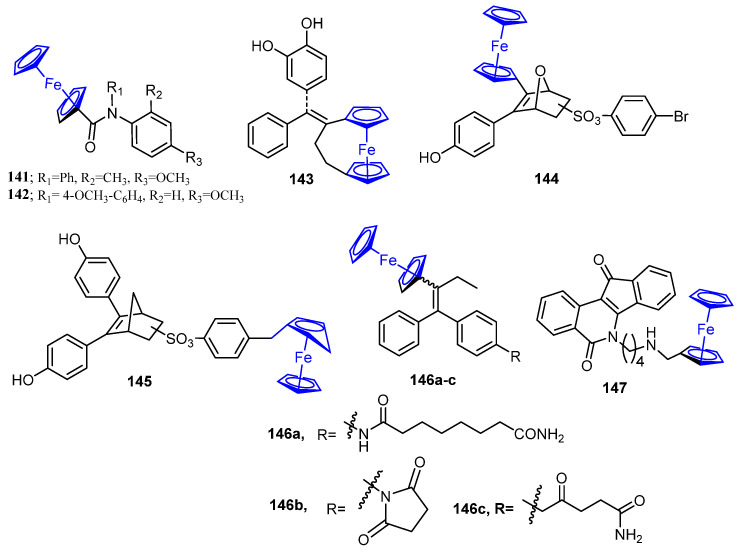
Miscellaneous compounds as anti-breast cancer agents.

On the other hand, Lu et al. developed eight novel ferrocenyl derivatives and assessed their in vitro anti-proliferative activity. The results showed that most of the synthesized compounds displayed good activity, in particular compounds **141** and **142** (Figure 20) exerted the best activity against MCF-7 (IC_50_ = 56 and 47 µM, respectively) and MDA-MB-231 (IC_50_ = 61 and 87 µM, respectively) cell lines [122].

In 2012, Tan et al. designed and synthesized a novel series of compounds formed by ferrocenyl group tethered to a catechol via a conjugated system. The compounds were assessed for their antitumor activity. Compound **143** (Figure 20) exhibited the highest anti-proliferative activity against the MDA-MB-231 cancer cell line with an IC_50_ value of 0.48 ± 0.04 µM [123]. Moreover, the Selective Estrogen Receptor Modulators (SERMs) bearing a ferrocenyl-oxabicyclo[2.2.1]heptenes were prepared and screened as anti-breast cancer agents. The results exhibited that compounds **144** (IC_50_ = 3.1 ± 0.5 µM for MCF-7) and **145** (IC_50_ = 7.8 ± 0.6 µM for MDA-MB-231) (Figure 20) were the most potent anti-proliferative agents against MCF-7 and MDA-MB-231 cancer cell lines [124].

Marinero and coworkers synthesized new derivatives of ferrocenyl compounds and evaluated their anticancer activity against TNBC MDA-MB-231 and MCF-7 cell lines. All these compounds displayed high antitumor activity with IC_50_ values ranging between 0.5 and 4.12 µM. Compounds **146a** and **146b** (Figure 20) exhibited the most efficient anti-proliferative activity toward the MDA-MB-231 cell line (IC_50_ = 0.50 µM and 0.54 µM, respectively), whereas **146c** (Figure 20) elicited the most potent activity against MCF-7 cells [125]. Furthermore, a novel class of indeno[1,2-*c*]isoquinolines bearing the ferrocenyl scaffold was synthesized. The most potent compound, **147**, (Figure 20) revealed the highest activity toward MDA-MB-231 breast cancer cell line compared to the reference drug etoposide [126].

**Figure 21:**
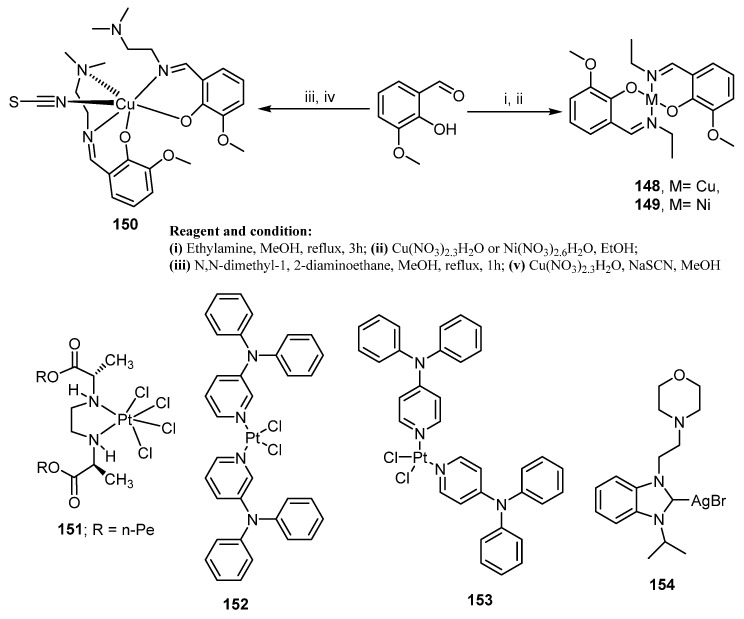
Organometallic complexes as anti-breast cancer agents.

Interestingly, organometallic complexes have taken reputation as effective and potent anticancer agents. Accordingly, three nano Ni(II) and Cu(II) complexes **148**–**150** (Figure 21) were synthesized via condensation reaction of 2-hydroxy-3-methoxybenzaldehyde with ethylamine or *N*,*N*-dimethyl-1,2-diaminoethane, followed by a complexation of the obtained Schiff base with Cu(NO_3_)_2_.3H_2_O and Ni(NO_3_)_2_.6H_2_O. The new complexes’ particle diameters were decreased using green manual grinding technology and the anti-proliferative activity of the produced complexes was assessed against the MCF-7 cancer cell line. The results displayed that complex **148** (Figure 21) revealed significant anticancer activity against MCF-7 cancer by inducing apoptosis [127].

In addition, it was reported that three novel platinum(IV) complexes were synthesized and screened for their anticancer activity. Compound **151** (Figure 21) showed the highest cytotoxic activity against MDA-MB-231 (IC_50_ = 68.023 µM) with induction of apoptosis [128].

Furthermore, two complexes of platinum(II) dichloride were synthesized and investigated against two breast cancer cell lines, MCF-7 and MDA-MB-231. Compounds **152** and **153** (Figure 21) showed potent inhibition activity and induced apoptosis in a similar way to cisplatin on tested cells. They also revealed anti-migration activity of MDA-MB-231 cells by decreasing the levels of metabolic energy (ATP), distressing cytoskeletal organization membrane and cell polarity [129].

In 2020, Kutlu et al. [130] investigated some synthesized Ag(I)-NHC complexes, having a morpholinoethyl and benzimidazole for their in vitro anti-proliferative activity against MCF-7 and MDA-MB-231 human BC cell lines. Compound **154** (Figure 21) (IC_50_ value of 17 ± 1.41 and 7.5 ± 0.77 µM against MCF-7 and MDA-MB-231 cell lines, respectively) displayed significant anti-proliferative activity, compared to cisplatin.

## 6. Conclusions and Future Perspective

Heading the list of critical health-associated problems worldwide, breast cancer stands as one of the most serious diseases responsible for a huge number of cancer-related mortality among women. Accordingly, development of novel efficient agents for management of the human breast malignancies is an urgent necessity. In this regard, considerable progress in the field of drug design and medicinal chemistry has been achieved, and several synthetic small molecules based on diversified chemical scaffolds have been identified as promising anti-breast cancer agents. This review summarized the recently reported different categories exploited to develop the synthetic anti-breast cancer candidates. To name just a few: quinoline, quinazoline, pyridine, pyridazine, pyrimidines, imidazole, benzimidazole, coumarin, tetrazole, indole, oxindole and triazine scaffolds were discussed in this review article. Moreover, the significant structure activity relationships, bioactivities and mechanistic insights for the reported small molecules were further concluded. It is worth stressing that surveying literature disclosed that there is a shortage in addressing invasion, migration and metastasis of BC, which reflects a discrepancy between the need and the assays for most of the reported anticancer agents. The present review article is expected to be contributory for the drug discovery community to support the future design and development of more potent, safer and selective candidates for breast cancer therapy.

## Figures and Tables

**Table 1:** Inhibition activities of quinazolines **18**–**21** against EGFR and HER-2 kinases, as well as against A549 and SK-Br3 breast cancer cell lines.

Comp.	*Enzyme Inhibition* IC_50_ (nM)		*Cell Growth Inhibition* IC_50_ (μM)	
	EGFR	HER2	A549	SK-Br3
**18**	8 ± 0.4	33 ± 0.10	2.03 ± 0.54	12.50 ± 2.41
**19**	10 ± 0.2	21 ± 0.7	3.60 ± 0.89	2.30 ± 0.37
**20**	20 ± 0.11	9 ± 0.10	1.22 ± 0.60	25.1 ± 8.54
**21**	19 ± 0.10	35 ± 0.8	4.49 ± 2.68	0.47 ± 0.35
**Lapatinib**	26 ± 0.12	17 ± 0.10	6.74 ± 1.33	0.49 ± 0.04
